# Introduction of a Variant Classification System for Analysis of Genotype-Phenotype Relationships in Heritable Retinoblastoma

**DOI:** 10.3390/cancers13071605

**Published:** 2021-03-31

**Authors:** Isabel Hülsenbeck, Mirjam Frank, Eva Biewald, Deniz Kanber, Dietmar R. Lohmann, Petra Ketteler

**Affiliations:** 1 Department of Pediatric Hematology and Oncology, University Duisburg-Essen, University Hospital Essen, Hufelandstrasse 55, 45122 Essen, Germany; Isabel.huelsenbeck@uk-essen.de; 2 Eye Oncogenetics Research Group, University Hospital Essen, 45122 Essen, Germany; deniz.kanber@uni-due.de (D.K.); diemar.lohmann@uk-essen.de (D.R.L.); 3 Institute for Medical Informatics, Biometry and Epidemiology, University Duisburg-Essen, University Hospital Essen, 45122 Essen, Germany; mirjam.frank@uk-essen.de; 4 Department of Ophthalmology, University Duisburg-Essen, University Hospital Essen, 45122 Essen, Germany; eva.biewald@uk-essen.de; 5 Institute of Human Genetics, University Duisburg-Essen, 45122 Essen, Germany; 6 German Cancer Consortium (DKTK), Partner Site Essen/Düsseldorf, 69120 Heidelberg, Germany

**Keywords:** tumor predisposition syndrome, heritable retinoblastoma, pRB, *RB1* gene, splice-site variants, classification, 13q-syndrome, screening

## Abstract

**Simple Summary:**

Heritable retinoblastoma is a genetic disease that predisposes to develop multiple retinoblastomas in childhood and other extraocular tumors later in life. It is caused by genetic variants in the *RB1* gene. Here we present a new classification for genetic variants in the *RB1* gene (REC) that focuses on the variant’s effect. The different classes, REC-I to -V, correlate with different risks of tumor predisposition. REC correlated with different clinical courses when applied in our study cohort. REC aims to facilitate risk estimation for physicians, patients and their families, and researchers and to improve the definition of the necessity of screening examination.

**Abstract:**

Constitutional haploinsufficiency of the *RB1* gene causes heritable retinoblastoma, a tumor predisposition syndrome. Patients with heritable retinoblastoma develop multiple retinoblastomas early in childhood and other extraocular tumors later in life. Constitutional pathogenic variants in *RB1* are heterogeneous, and a few genotype-phenotype correlations have been described. To identify further genotype-phenotype relationships, we developed the retinoblastoma variant effect classification (REC), which considers each variant’s predicted effects on the common causal mediator, *RB1* protein pRB. For validation, the *RB1* variants of 287 patients were grouped according to REC. Multiple aspects of phenotypic expression were analyzed, known genotype-phenotype associations were revised, and new relationships were explored. Phenotypic expression of patients with REC-I, -II, and -III was distinct. Remarkably, the phenotype of patients with variants causing residual amounts of truncated pRB (REC-I) was more severe than patients with complete loss of *RB1* (REC-II). The age of diagnosis of REC-I variants appeared to be distinct depending on truncation’s localization relative to pRB structure domains. REC classes identify genotype-phenotype relationships and, therefore, this classification framework may serve as a tool to develop tailored tumor screening programs depending on the type of *RB1* variant.

## 1. Introduction

Heritable retinoblastoma is a rare autosomal dominant tumor predisposition syndrome caused by constitutional haploinsufficiency of the *RB1* gene [1]. About 20–30 children heterozygous for pathogenic *RB1* gene variants are diagnosed each year in Germany. Most of these children develop multiple retinoblastomas in their first years of life, either in one (unilateral) or in both eyes (bilateral). Mutational inactivation of the second *RB1* allele initiates the development of these tumors. Patients with heritable retinoblastoma also have a predisposition to develop extraocular cancers, mainly sarcoma, which most often develop later in life. *RB1* may be regarded as the prototype tumor-suppressor gene with high penetrance in a specific age range, but genotype-phenotype correlations are very complex, as shown in previous studies [2,3,4,5,6].

Retinoblastoma is an embryonal tumor, and findings indicate that the cell-of-origin is a cone photoreceptor precursor [7]. Retinal development is completed in early childhood, explaining why retinoblastoma is rarely diagnosed after five years. A model that explains why the haploinsufficiency of the *RB1* gene confers a high risk to tumorigenesis, specifically to these cone precursor cells, is still missing.

**Figure 1:**
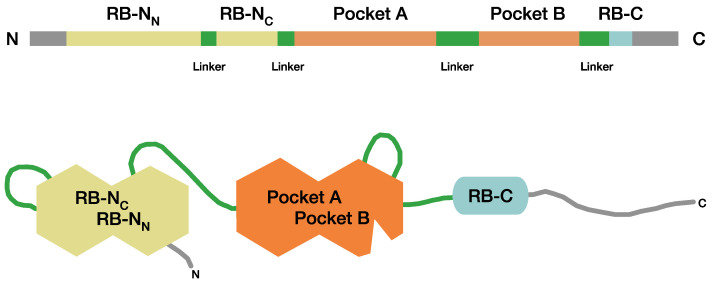
Protein domains of pRB. Schematic image of pRB according to Dick and Rubin [10,16] showing the structural domains RB-N, pocket A, pocket B, and RB-C. Linker regions are displayed as red lines.

The *RB1* gene codes for pRB, which is an almost ubiquitously expressed phosphoprotein of 928 amino acids. Loss of functional pRB leads to a response that alters the cell type-specific signaling circuitry, which helps to drive tumorigenesis of retinoblastoma [8,9]. In cells with normal *RB1* alleles, dephosphorylated pRB binds E2F-transcription factors and blocks transition from G1- to S-phase. Phosphorylation of pRB via cyclin D and CDK4/6 regulates the cell cycle transition into S-phase. Cells expressing non-functional pRB can show uncontrolled proliferation and possible tumorigenesis [10]. A distinctive structural feature of pRB is the pocket domain, which consists of two subdomains, A and B, that can bind E2F transcription factors [11] (Figure 1). The pocket region sequence has high evolutionary conservation and similarity with the pocket domains of the other two retinoblastoma protein family members, p107 (RBL1) and p130 (RBL2). These two proteins are paralogs of *RB1* present in all higher eukaryote genomes, and their functions in regulating growth, the cell cycle, and E2F function are analogous [10]. In mice, loss of one of the other RB pocket proteins, p107 and p130, and loss of pRb are required to develop retinoblastoma [12]. The other main structural domains of pRB are the bipartite amino-terminal region RB-N (RB-N_C_ and RB-N_N_) and the carboxyterminal region RB-C, which both show lower sequence conservation [13,14]. RB-N_C_ and RB-N_N_, pocket A and B, and the RB-C domain form alpha-helical structures connected by linker regions containing CDK-dependent target sites for phosphorylation that have a critical role for the functional state of pRB [13,14,15] (Figure 1). Phosphorylation at the linker within RB-N and at the linker region between RB-N and pocket A leads to the association of RB-N to the pocket. Phosphorylation at the linker within the pocket (between pocket A and pocket B) induces association of the linker (pocket-loop) to the pocket and phosphorylation at the linker between pocket B and RB-C results in the association of RB-C to the pocket [10,16]. In summary, posttranslational modification of pRB facilitates *cis* interactions of regions within this protein, and the resulting distinct protein conformations determine the functional state of pRB.

Various alterations of the *RB1* gene sequence can cause heritable retinoblastoma (http://rb1-lsdb.d-lohmann.de (accessed on 1 February 2021)). It appears that all pathogenic *RB1* variants ultimately result in quantitative or structural alterations of pRB. Most patients with heritable retinoblastoma are heterozygous for variant alleles that create premature termination codons (PTCs) in regions of the *RB1* gene where PTCs are expected to cause nonsense-mediated decay (NMD) [17,18]. Consequently, it is plausible that levels of variant mRNA from these alleles are diminished compared to mRNA levels from normal alleles. Furthermore, any transcripts evading NMD are translated into a C-terminal truncated pRB. *RB1* variants that result in missense and in-frame alterations are expected to result in structural alterations of pRB. The pathogenic variants identified in the *RB1* promoter region’s regulatory elements will result in reduced expression of a normal pRB. Less than 10% of patients with heritable retinoblastoma have large deletions that include the whole *RB1* gene, and a complete absence of pRB expression is to be expected. Large deletions often involve neighboring genes on chromosome 13q, and this loss may modify phenotypic expression of retinoblastoma predisposition [19,20].

Phenotypic expression of heritable retinoblastoma has several features. Observed variables include laterality of retinoblastoma, age and tumor stage at initial diagnosis, and, in some patients, the number of independent tumor foci. Some patients also show retinoma, which are essentially benign retinal lesions, or develop embryonic midline tumors such as pineoblastoma [21,22,23]. Patients with heritable retinoblastoma have a higher risk of extraocular malignancies (second primary malignancies, SPM) later in life compared to children without constitutional *RB1* variant [24,25,26]. The spectrum of phenotypic expression of heritable retinoblastoma includes incomplete penetrance, which is defined as the absence of retinoblastoma or retinoma in an adult who is heterozygous for a pathogenic *RB1* allele. Overall, incomplete penetrance is rare, but some families show a conspicuous aggregation of unaffected carriers. This particular phenotype distribution within families, which is often referred to as low-penetrance retinoblastoma, is associated with a subset of pathogenic *RB1* variants [27,28]. These variants appear to have in common that they either result in reduced levels of expression of a normal pRB or in structural alterations of pRB that diminish only some of its diverse functions (class I and II according to Otterson [2] reviewed by Harbour [29]). However, it has not been resolved which functions of pRB are critical for this genotype-phenotype relationship.

The initial observation in two families that penetrance of heritable retinoblastoma can depend on the parental origin of the variant allele has recently been supported by additional observations of parent-of-origin effect in other families with low-penetrance retinoblastoma [4,5,30]. As the *RB1* is an imprinted gene, maternal and paternal alleles are not identical, but a plausible model explaining why phenotypic expression is sensitive to the variant allele’s parental origin is still missing. [31,32]. Another genotype-phenotype association has been described in patients with large deletions. Depending on the genes that are lost in addition to *RB1*, these patients show additional phenotypic features such as psychomotor delay and a characteristic craniofacial dysmorphism [33]. Carriers of large deletions tend to develop fewer tumor foci if the deletion affects neighboring genes that are critical for cell viability, such as the *MED4* gene [19,20]. Consequently, *MED4* is a modifier gene located in *cis* to the *RB1* gene. Tumor predisposition in heritable retinoblastoma is also influenced by modifier genes in *trans*, and a known example is a polymorphic variant in the MDM2 promoter [6].

Finally, patients with somatic mutational mosaicism, which is present in a considerable fraction of patients with isolated retinoblastoma, are known to develop fewer tumor foci compared to patients heterozygous for a pathogenic *RB1* variant [34,35,36]. It has been proposed that the influence of mosaicism on phenotypic expression “depends on the nature of the first mutation, its time in development, and the number and types of cells that are affected” [37,38]. The level of mosaicism in DNA from blood may serve as a proxy parameter [39]. For the purpose of the analyses of genotype-phenotype associations presented here, we did not include patients with known somatic mosaicism.

The above-mentioned genotype-phenotype relationships are only informative in a small percentage of patients. For most *RB1* variants, observational data from other patients are not available, and, therefore, individualized prediction of tumor risk is not possible. The growth of knowledge about genotype-phenotype associations is slow because the number of patients in each country is low, and there is limited phenotype data for individual *RB1* variants [40]. We sought to improve this situation by defining *RB1* variant classes that may show similar phenotypic outcomes. This classification concept assumes that the phenotypic effects of pathogenic *RB1* variants are mediated solely via variation of structure or amount of pRB. In other words, pRB is the last known mediator toward phenotypic expression (see [41] for a short introduction to causal inference). Classification of genetic variants based on the effect on proteins does not exist for *RB1* variants but has been described for other genetic diseases, i.e., cystic fibrosis (CF) [42]. In some populations, CF is a relatively frequent autosomal recessive disease caused by pathogenic variants in the *CFTR* gene that codes for a transmembrane chloride channel protein. Pathogenic variants cause dysfunction of this protein, and this leads to the clinical picture of chronic pulmonary obstruction, pancreatic enzyme insufficiency, and reduced fertility in males [42]. About 70% of patients with CF show a single amino acid deletion (F508), but multiple other disease-causing variants in the *CFTR* gene have been described [43]. These variants have been grouped into five general classes that correspond with the type of protein alteration and the dysfunction of the chloride channel. This variant classification is used to predict the phenotypic outcome expected in a patient with a specific pair of variant alleles and to adjust diagnostic and treatment decisions accordingly [43].

Here, we present a new classification for pathogenic *RB1* variants that is based on the effect of each *RB1* variant on alteration of pRB amount, structure, and function. It extends the established two categories that were introduced to explain incomplete penetrance [2] and includes assumptions well justified by current knowledge of transcription and translation of variant alleles. Using this prior knowledge, we aim to increase the homogeneity of features within groups of patients sharing variants of the same retinoblastoma variant effect classification (REC) class. For validation of this classification, we analyze data from a retrospective cohort of patients with heritable retinoblastoma in respect to this classification. REC could be a useful tool to interpret the potential phenotype of variants for attending physicians.

## 2. Materials and Methods

### 2.1. Patient Cohort

**Figure 2:**
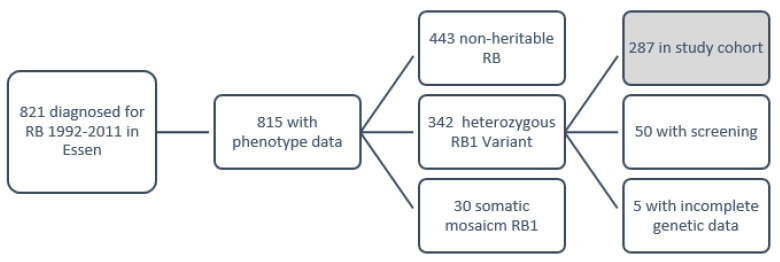
Modified consort diagrams of inclusion of patients.

A cohort of 821 national and international patients with diagnoses of retinoblastoma and genetic analysis between 1992 and 2011 at the German referral center were screened for the retrospective study cohort (see modified consort diagram in Figure 2). Six patients were excluded because of incomplete phenotype data. Data of genetic analysis showed no evidence of pathogenic constitutional *RB1* variant in 443 patients (54.4% of 815 patients) (tumor and blood DNA available in 332 patients, only blood DNA available in 108 patients, 3 without *RB1* variants characterized by high MYCN amplification) and somatic *RB1* mosaicism in 30 patients (3.7% of 815 patients). Of 815 patients, 342 had a heterozygous constitutional pathogenic *RB1* variant (41.9%), but 5 patients had incomplete genetic data. Of the remaining 337 patients, 50 patients were excluded from the study cohort because they received ophthalmological screening examinations for familial retinoblastoma. In total, the study cohort consisted of 287 patients with proven constitutional heterozygous pathogenic *RB1* variant that did not receive screening for retinoblastoma. The study cohort included 39 patients with familial retinoblastoma who did not receive presymptomatic screening (13.6% of 287 patients), 245 (85.4%) with sporadic heritable retinoblastoma, and 3 patients without information on family history (1.0%). The most common reasons for the absence of ophthalmological screening in familial retinoblastoma were missing or misinterpreted information of the affected parent about the heritability of retinoblastoma and the need for screening the offspring. Ethics approval was obtained from the ethics committee of the University of Duisburg-Essen (no: 15-6333-BO).

### 2.2. Data Acquisition

Clinical data on retinoblastoma were collected from the medical records. Genetic testing was performed on blood and tumor tissue at the request of individuals or their legal guardians with the aim to identify the oncogenic alterations of the *RB1* gene. Molecular genetic variant identification analysis was performed on DNA from fresh-frozen tumor samples or DNA from blood as reported previously [34,44,45,46]. This included one or more of the following methods: analysis of allele loss in tumors, cytogenetic analysis, denaturing high-performance liquid chromatography, exon-by-exon sequencing, multiplex ligation-dependent probe amplification, methylation-sensitive PCR, quantitative fluorescent multiplex PCR, quantitative real-time PCR, and single-strand conformation polymorphism. Diagnosis of mutational mosaicism was based on the finding of a skewed signal ratio of the variant to the normal allele relative to the ratio obtained from heterozygous DNA.

### 2.3. Data Analysis

The data were retrieved and subjected to statistical analyses with IBM SPSS Statistics version 27.0 (Chicago, IL, USA) and R version 4.0.3 (packages: tidyverse collection, haven, survival, survminer, ggmosaic). Some of the analyses were explorative, and this essentially precludes a truthful control for multiple testing and the definition of a cut-off for significance. Nonetheless, statistical analysis was performed to compare the phenotypic features between the groups. Specifically, distribution of age at diagnosis was compared with exponential model analysis using the survreg function of the survival package in R (https://cran.r-project.org/package=survival (accessed on 1 February 2021)). The annotation of the different domains of the protein structure was based on the description by Dick and Rubin [10].

### 2.4. A System to Classify Pathogenic RB1 Gene Variants for Analysis of Genotype-Phenotype Relationships

Various genetic alterations of the *RB1* gene can cause heritable retinoblastoma. Despite some recurrent variants, each individual variant is rare. To facilitate the identification of genotype-phenotype relationships, combining observations concerning different variants is required. We devised a system for grouping variants that is inspired by concepts of causal inference (retinoblastoma variant effect class, REC). The underlying idea is that any effect of variant alleles on phenotypic expression is mediated by alterations of pRB. Therefore, if genotype-phenotype relationships are to be analyzed, it is rational to group distinct pathogenic variants with similar effects on pRB structure and quantity. Applying these principles, the majority of pathogenic alleles can be classified in one of 5 main classes (Table 1, Appendix A, Figure A2). Variants resulting in PTCs that elicit NMD belong to REC-I, which is defined by residual expression of a C-truncated pRB. Several distinct types of DNA alterations are expected to have a REC-I effect, including small variants in exons 2 to 25 of the *RB1* gene as well as alterations of invariant splice signals that result in variant mRNAs with PTCs. REC-II summarizes all variants that result in complete loss of pRB and comprises whole *RB1* gene deletions and partial deletions that include the promoter region. REC-III contains alterations that result in altered mRNA without PTCs and, thereby, in a pRB with a focal structural change only. Variants in the open reading frame (ORF) part of exon 1 that introduce PTCs appear to result in variant mRNAs evading NMD [17]. Moreover, case reports support that downstream translation initiation may occur and result in a variant pRB lacking some N-terminal sequence [47], which is a focal structural change. Although it is not resolved if these consequences are general features of exon 1 variants that result in PTCs, we classified these variants in REC-III. It is assumed that the effect of variants at remote splice-sites on splicing can be leaky, i.e., a proportion of variant primary transcripts are processed in a normal mRNA. All variants located in splice-sites are grouped into either REC-I variants or REC-III variants. Depending on the localization of the variant within the splice-site, the effect varies from leaky to complete, which has to be determined for each variant. This justifies the separation of REC-I-leaky and REC-III-leaky from the main classes in the functional *RB1* genotype classification. To allow for this, REC-I-leaky and REC-III-leaky are used depending on the presence or absence of PTCs in the predicted variant mRNA, respectively. REC-IV and REC-V represent effects expected from 3′-end deletions and regulatory promoter variants, respectively. In summary, the proposed classification system for genotype-phenotype relationships of *RB1* variants integrates previously published knowledge on the consequences of variants on pRB structure and quantity and on phenotypic expression.

## 3. Results

### 3.1. Clinical and Genetic Characteristics of the Study Cohort

**Figure A1:**
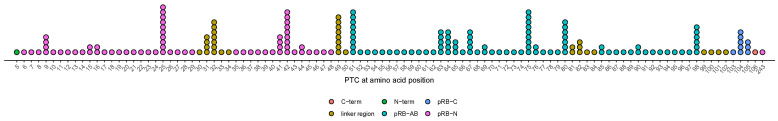
Localization of *RB1* variants resulting in PTC according to the pRB-structures. REC-I variants were localized according to the position of the stop codon. PTC, premature termination codon.

Clinical and genetic characteristics of the 287 patients with constitutional heterozygous pathogenic *RB1* variants in our retrospective study cohort are presented in Table 2. The number of patients with *RB1* variants in the distinct classes is summarized in Table 3. Variants were scattered across the entire gene and transcription factor binding site in the promoter region. Small alterations expected to result in PTC showed a local distribution with prominent recurrences of transitions at methylated CpG dinucleotides that result in a PTC (Appendix A, Figure A1). Exons 5 and 26 showed no small alteration, but these regions are rather small and do not contain CpG-transition recurrence sites.

### 3.2. Most Patients in the Study Cohort Are Heterozygous for Variants That Result in PTCs Expected to Elicit NMD (REC-I)

**Figure A2:**
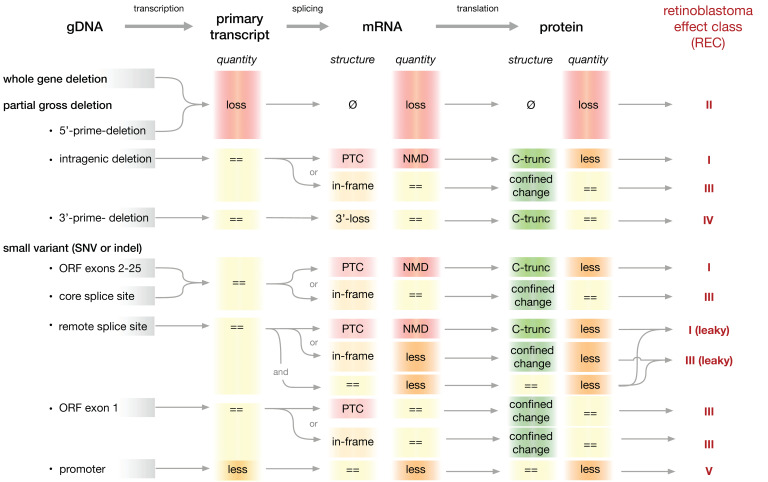
Retinoblastoma effect classes (REC). *RB1* variants were grouped into 5 REC classes and 2 subgroups (REC-I-leaky, REC-III-leaky) according to the effect on pRB. PTC, premature termination codon; NMD, nonsense-mediated decay; ORF, open reading frame; C-trunc, C-truncation of pRB.

The constitutional pathogenic *RB1* variants in the study cohort were classified according to their effect on pRB as explained in the method sections (Table 1 and Table 2, Appendix A, Figure A2). REC-I variants (at least partial elimination of transcripts by NMD and expression of C-truncated pRB from the remaining mRNA) were detected in 199 children (69.3%), and 39 children (13.6%) were heterozygous for variants that plausibly cause complete loss of pRB (REC-II variants). REC-II variants were subgrouped into deletions, including the *MED4* gene (REC-II+, 27 of 39 patients) and deletions without deletion of *MED4* (REC-II−, 6 of 39 patients). Information on deletion size was missing in six patients. REC-III variants (confined alteration of pRB structure) were found in 45 children (15.7% of 287 patients). Seven of these 45 patients were heterozygous for variants that result in the expression of a pRB expected to lack part of the N-terminus. Only one child (0.3%) was heterozygous for a variant of REC-IV (C-truncation of pRB without quantitative changes), and three children were heterozygous for variants (1.0%) in the promoter-associated regulatory elements expected to result in diminished expression of pRB compared to normal (REC-V). The cohort included 66 patients (23.0%) with splice-site variants. Most of these variants altered invariant splice-site positions (*n* = 51, 77.3%) and were grouped in REC-I or REC-III depending on the expected effect on the ORF within the variant mRNA. Variants in 15 patients (22.7% of all variants localized at splice-sites) altered less conserved positions at splice-sites. For some of these variants, the effect on splicing had been validated by previous transcript analysis [46]. It is plausible to assume that the effect of at least some of these variants on splicing is leaky.

### 3.3. Phenotypic Expression in Patients Heterozygous for Variants Previously Associated with Incomplete Penetrance

Incomplete penetrance (IP) of retinoblastoma has been observed in some families with heritable disease, and this phenomenon is, at least in part, due to the functional effect of some variants. In our study cohort, 47 patients had variants that were previously observed in at least one heterozygous individual without retinoblastoma (Appendix A, Table A1). These include two patients with a variant of subset REC-I-leaky and 27 patients with whole *RB1* deletions that extend into the *MED4* gene (subset REC-II+). Most variants with observed association to incomplete penetrance were classified as REC-III (15 REC-III-non-leaky, 1 REC-III-leaky). Two patients carried REC-V variants (Appendix A, Table A1). Comparison of the phenotype of patients with IP variants to patients with variants without observed association with IP showed a lower percentage of bilateral retinoblastoma (63.8% and 93.8%, respectively) and a similar age at diagnosis (median: 12.4 months (range: 0.4–40.9 months) and 8.0 months (0.2–48.0 months)) (Table 4).

### 3.4. Patients with REC-I Variants Show Earlier Age at Diagnosis and More Tumor Foci Compared to Patients with Variants of Other Effect Classes

**Figure 3:**
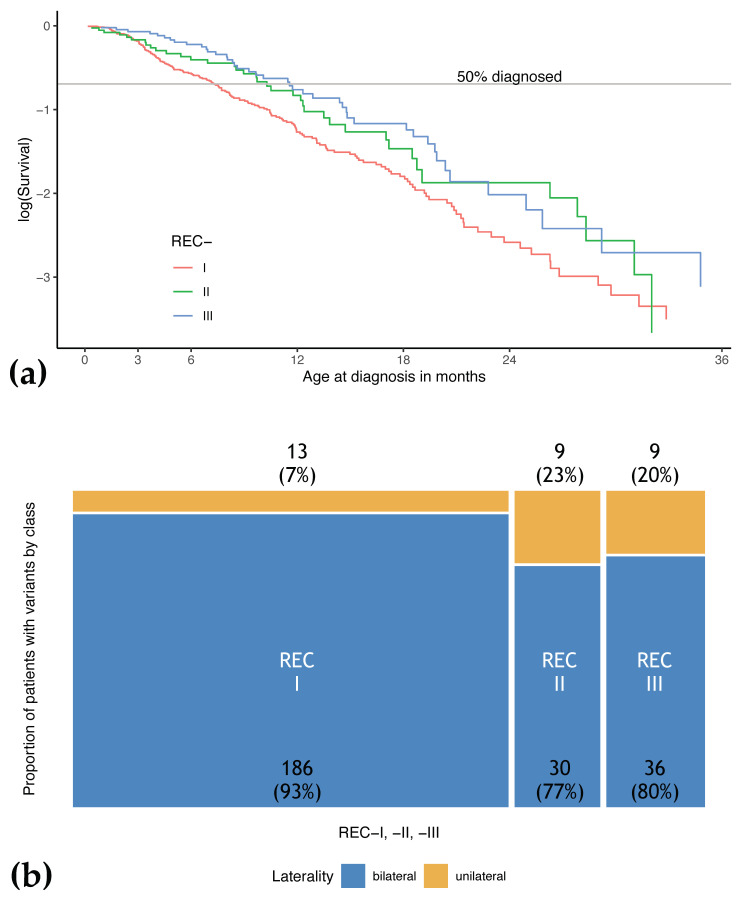
Phenotype of patients heterozygous for *RB1* variant depending on REC-I, -II, and -III. (**a**) Analysis of age at diagnosis of patients with REC-I,-II, and -III variants shows that the risk of initial diagnosis of retinoblastoma at a given age in a child with a REC-I variant is higher compared to a child with a REC-II or -III variant (32% increased risk, *p* = 0.03, exponential model). (**b**) Distribution of laterality in patients REC-I, -II, and -III variants displayed in a mosaic plot.

The distributions of phenotypic expression in patients with REC-I (NMD and expression of C-truncated pRB), -II (complete loss of pRB), and -III variants (confined alteration of pRB structure) were compared. REC-IV and -V variants were not included at this stage due to the low number of observations (one and three patients, respectively). The percentage of bilateral retinoblastoma was higher in REC-I (93.5%) than in REC-II (76.9%) and REC-III (80.0%) (Figure 3b). The age of diagnosis was lowest in patients with REC-I variants (median: 7.3 months [range: 0.2–48.0], followed by REC-II (10.3 months, [0.4–40.9]) and REC-III (11.6 months [0.9–45.1]). According to Knudson’s hypothesis, the fraction of patients with heritable retinoblastoma not yet diagnosed (S) follows an exponential distribution (logS~t, Figure 1 in Knudson 1971 [48]). In fact, plotting logS versus age at diagnosis of patients with REC-I,-II, and -III variants resulted in almost linear distributions as expected under an exponential model. It appeared that the slopes of these distributions, which correspond to the rate of events, are distinct between REC-I, -II, and -III (*p* = 0.03) (Figure 3a).

### 3.5. Phenotype of Patients with REC-II Variants Depends on Involvement of MED4

**Figure 4:**
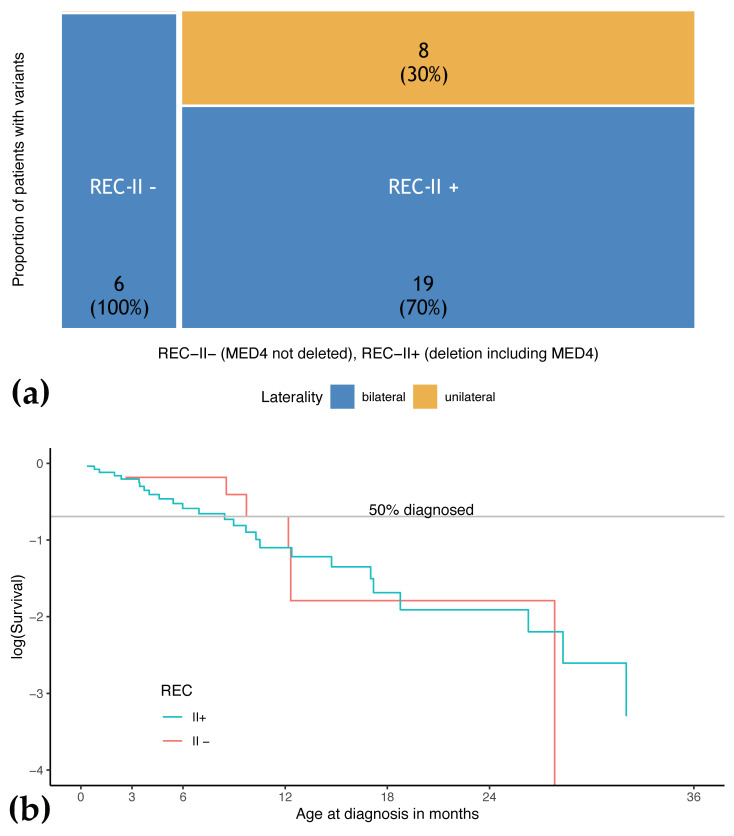
Distribution of phenotypic criteria in patients REC-II+ in comparison with REC-II–(a) (**a**) Distribution of laterality in patients with REC-II− and REC-II+ variants displayed in a mosaic plot for laterality. (**b**) Analysis of age at diagnosis in patients with REC-II− and REC-II+.

All large *RB1* deletions that result in complete loss of pRB expression from this allele were classified as REC-II variants. However, these variants vary in size ranging from deletions of the *RB1* gene only to deletions of multiple genes on chromosome 13q. It is established that the mean number of eyes with retinoblastoma in patients with large deletions is distinct depending on the loss of some adjacent genes, specifically the *MED4* gene [20]. In the study cohort, 27 of the 39 REC-II variants (69.2%) included the *MED4*-gene (REC-II+), six patients (15.4%) carried deletions of the whole *RB1* gene without the involvement of *MED4* (REC-II−), and in six patients (15.4%), the extent of the deletion outside of the *RB1* gene was not characterized in sufficient detail. All six REC-II− patients developed bilateral disease, while only 19/27 (70.4%) of REC-II+ patients developed bilateral disease. The distribution of age of diagnosis was similar (REC-II−: 11.0 months (2.6–27.8), REC-II+: 8.4 months (0.4–40.9)). The logarithmic plot of the age of diagnosis shows overlap but suggests a different pattern of distribution of age at diagnosis between REC-II− and REC-II+ (Figure 4) (*p* = 0.38). The phenotypic expression of patients with REC-II− than that of patients with REC-I variants (median age at diagnosis: REC-II–: 11.0 months (2.6–27.8) vs. REC-I: 7.2 months, REC-II−: 100.0% bilateral vs. REC-I: 93.5% bilateral). However, it has to be pointed out that the number of observations in the REC-II− class is small.

### 3.6. Patients with REC-III Variants Show a Broad Variety of Phenotypic Expression

**Figure 5:**
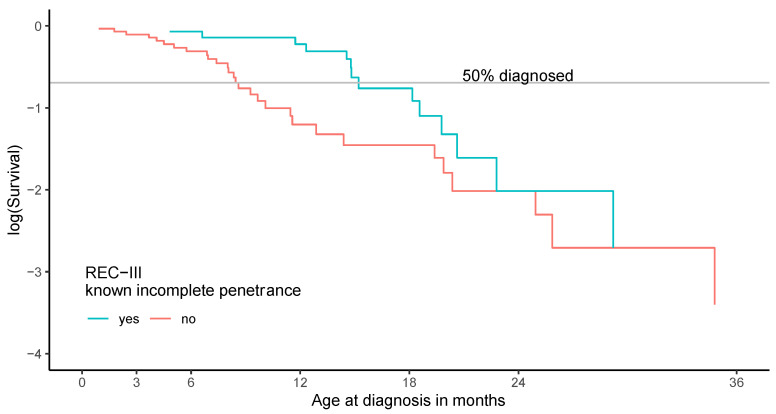
Age at diagnosis of REC-III of *RB1* variants in patients heterozygous for *RB1* variant. Age at diagnosis of patients with REC-III variants shows a trend for a higher age at diagnosis in patients with documented incomplete penetrance compared to those without documented incomplete penetrance (*p* = 0.23, exponential model).

Fifteen of 45 patients in REC-III (confined alteration of pRB structure) (33.3%) carried variants that have been previously described in families with incomplete penetrance (Appendix A, Table A1). In this group of patients, less bilateral retinoblastoma occurred (IP: 66.7% vs. no IP observed: 90.0%) compared to those without reports on incomplete penetrance. Analysis of the distribution of age at diagnosis (logS~age at diagnosis) of patients with REC-III variants showed a trend for a higher age at diagnosis for the subgroup with established IP variants (IP: median 15.2 (4.8–39.3) vs. no IP observed: median 8.5 (0.9–45.1), *p* = 0.23) (Figure 5). Patient numbers in REC-III were too small to further stratify phenotype distributions by different localizations of focal changes within pRB.

### 3.7. Age at Diagnosis of Patients with Variants Resulting in PTCs in Unstructured Linker Regions Appears to Be Earlier Compared to Patients with PTC in Other Regions

**Figure 6:**
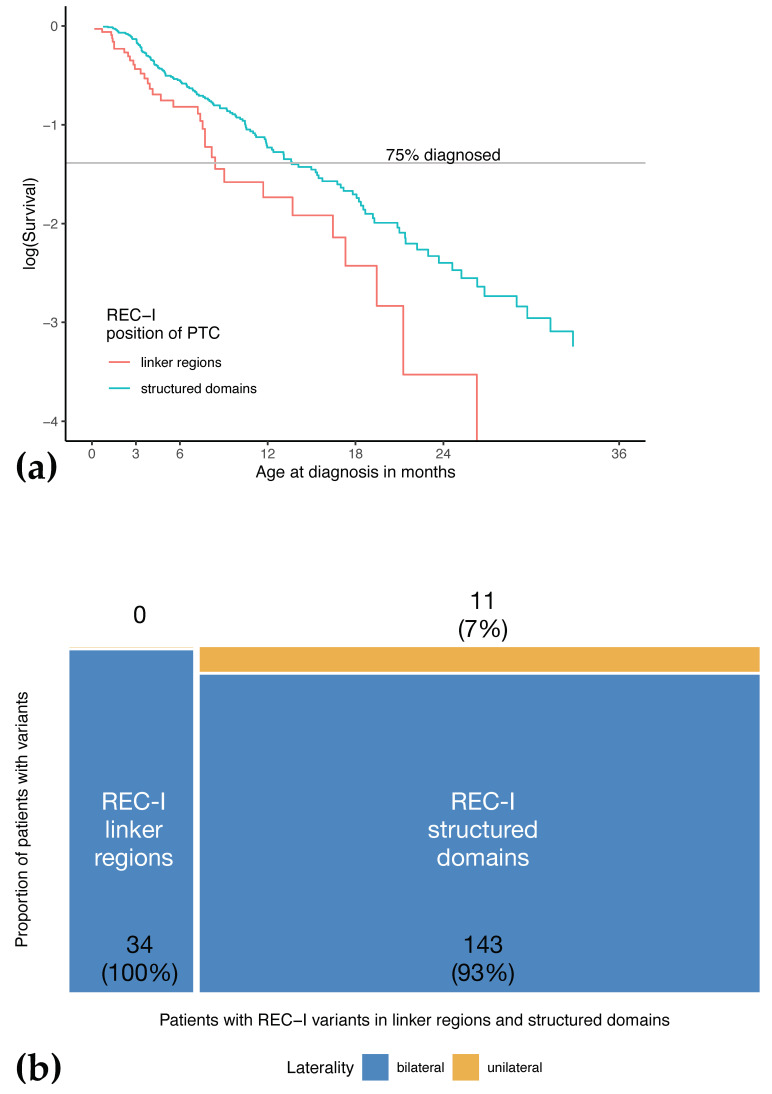
Age at diagnosis was lower in patients with PTC variants affecting the linker regions. (**a**) Analysis of age at diagnosis of patients with REC-I variants in pocket A, pocket B, and RB-N (structured) compared to REC-I variants in linker regions (unstructured) shows that the risk of initial diagnosis of retinoblastoma at a given age in a child with a REC-I linker region variant is higher compared to a child with a REC-I variant located in a structured region (49% increased risk, *p* = 0.037 exponential model). (**b**) Distribution of laterality in patients with REC-I variants in a mosaic plot shows that all PTC in linker regions (*n* = 34) caused bilateral disease. The majority of premature termination codons (PTCs) in structured regions (*n* = 165) also caused bilateral disease, but 7% remained unilateral.

Patients with variants resulting in at least partial NMD and expression of a C-truncated pRB are summarized in REC-I. Elimination of transcripts with PTCs by NMD is not complete. The mutant pRB that is translated from mRNAs that have evaded NMD is expected to show truncation of C-terminal parts of the protein depending on the location of the PTC. As our cohort of patients included numerous patients with REC-I variants, it was feasible to analyze if the phenotypic expression is distinct by the location of the PTC. Bins of PTC locations were defined according to the known regions of pRB as described in detail in the review article by Dick and Rubin [10]. PTCs of REC-I variants observed in our study cohort were found in all structural domains of the pRB (Appendix A, Figure A1). The distribution of PTC is not uniform, but this is to be expected from the presence of several mutation-prone sequence motifs within the *RB1* gene, such as methylated CpG dinucleotides. When exploring relationships between age at diagnosis and PTC location, we observed that all nine patients with a PTC located in the small region of pRB that forms a linker between RB-N and pocket A showed an early age at diagnosis with a median of 1.5 months (0.2–9.0) compared to 7.6 months (0.7–48.0) in patients with variants resulting in truncation in other parts of the pRB. In order to test if this genotype-phenotype relationship may represent a regular feature, we generalized from this specific finding and analyzed if patients with PTCs in any of the linker regions outside structured domains may be associated with a phenotypic expression distinct from that of PTCs in one of the structured domains, namely pocket A, pocket B, and RB-N. The plotted data appear to fit Knudson’s assumption of an exponential distribution. The slopes of the two groups, PTCs in linker vs. structured regions, were distinct, and this supports the hypothesis that age at diagnosis depends on PTC location (*p* = 0.037) (Figure 6a). A difference in phenotypic expression depending on PTC location was also apparent in laterality: all 34 patients with truncation in a linker region eventually developed bilateral disease (Figure 6b).

### 3.8. Effect of Alterations at Splice-Sites Depending on Their Relative Location

Patients with REC-I-leaky variants (*n* = 9) showed a trend for higher age at diagnoses and less often bilateral presentation than patients with patients in REC-I-non-leaky (*n* = 190) (age at diagnosis: REC-I-leaky: 12.1 months (2.5–19.3 months) vs. REC-I-non-leaky: 7.2 months (0.2–48.0), *p* = 0.77, percentage of bilateral disease: REC-I-leaky: 77.8% vs. REC-I-non-leaky: 94.2%). Of note, some of the patients with REC-I-leaky variants developed a full phenotype similar to that of patients with regular REC-I variants. No differences in age at diagnosis or laterality were observed between patients with REC-III-non-leaky and REC-III-leaky variants.

## 4. Discussion

Identification of genotype-phenotype relations for heritable retinoblastoma has been addressed by others and our own group before, but many questions remain unanswered [3,49,50]. The classification approach presented here summarizes the diverse types and consequences of pathogenic variants of the *RB1* gene and may serve as a conceptual framework for the identification of genotype-phenotype relationships. Specifically, improving the estimation of age-dependent risk for additional tumors in patients with heritable retinoblastoma can improve the prediction of the clinical course of affected individuals and enables the attending physicians to tailor the clinical management and screening intervals. Moreover, deeper knowledge about genotype-phenotype relationships could further elucidate how functional alterations of pRB contribute to the tumorigenesis of retinoblastoma.

The spectrum of pathogenic *RB1* variants is very broad, as reflected in our retrospective study cohort comprising children diagnosed with heritable retinoblastoma between 1992–2011 and in other national cohorts [38,49,50,51]. At the same time, phenotypic expression in individuals with heritable retinoblastoma features a wide range of criteria such as age at diagnosis, tumor stage at diagnosis, laterality, number of tumors in each eye, and incidence and type of SPM. Genotype-phenotype relationships in heritable retinoblastoma have been described for families with low-penetrance retinoblastoma [52]. The defining feature of families with low penetrance is the occurrence of incomplete penetrance in heterozygous family members [2]. It has been confirmed by many groups that low-penetrance retinoblastoma is associated with variants resulting in lower-than-normal levels of expression of an unaltered pRB or an expression of a pRB with alterations confined to some regions [5,20,29,49]. In our study cohort, patients with small variants previously associated with incomplete penetrance of retinoblastoma showed a lower proportion of bilateral manifestation and higher age at diagnosis compared to patients with variants without observed instances of incomplete penetrance.

*RB1* variants associated with incomplete penetrance are rare. Little is known whether there is genotype-dependent heterogeneity of phenotypic expression also among patients with variants that are regularly associated with complete penetrance. Individuals heterozygous for variants expected to trigger NMD (REC-I) usually show complete penetrance of retinoblastoma [38,50,53]. However, the efficiency of NMD is incomplete, and it is plausible that these variant alleles result in residual expression of a C-terminal truncated pRB. It has been recognized in early studies on genotype-phenotype relationships that variants that fall into this class are associated with a severe phenotype, including numerous tumors in both eyes [54]. This has been confirmed by recent larger studies. In the Dutch national cohort including 126 nonfamilial patients with heterozygous *RB1* variants, children with frameshift and nonsense variants (most of these correspond to REC-I variants) developed more bilateral retinoblastoma than children with missense variants (REC-III) or large chromosomal deletions (REC-II) [49]. Recently, data of a large French national cohort of 512 children with constitutional *RB1* variants were analyzed for associations between genotype and phenotype [3]. The authors compared variants associated with presence to those associated with the absence of pRB and, for analysis of laterality, further subcategorized variants by the type of DNA alteration. Patients with variants associated with absent pRB (most correspond to REC-I and REC-II variants) showed a higher proportion of bilateral disease (absent pRB 84.2%, altered pRB 65.2%, respectively) and younger age at diagnosis (absent pRB mean age 12.3, altered pRB 16.3 months, respectively) than children with variants associated with an altered but present pRB (most correspond to REC-III variants) [3]. Moreover, the subcategory of large deletions or duplications associated with the absence of pRB showed a significantly lower proportion of patients with bilateral disease (73.8%) compared to 89.2% in patients with nonsense variants which is in accordance with the findings of the Dutch national cohort [3,49]. These subcategories, large deletions and nonsense variants, correspond to REC-II and REC-I in our classification system, and data from patients in our study group confirm this genotype-phenotype relationship (proportions of bilateral disease: REC-II: 76.9% and REC-I: 93.5%).

It has been established that the phenotype associated with loss of the *RB1* gene (REC-II) is milder if the deletion includes some adjacent genes, specifically *MED4* [19,20,44]. In agreement with this relationship, we observed less bilateral manifestation in patients in REC-II+ compared to those in REC-II−. However, children with *MED4* deletion were diagnosed at a similar age as children without *MED4* deletion. The feature age at diagnosis may be confounded because children with large deletions may show clinical signs associated with a 13q- deletion syndrome, including dysmorphic features, developmental delay, and failure to thrive. These children may receive more medical attention than average, with an increased likelihood of early detection. After excluding children with additional deletion beyond *MED4*, the phenotypic expression of patients with complete loss of *RB1* without the involvement of *MED4* (REC-II−) was still milder than that of patients with REC-I variants.

Our classification concept does not assume that REC-I variants result in complete loss of pRB. It takes into account that NMD of transcripts of variant *RB1* alleles with PTCs in exons 2 to 25 is incomplete and thus allows for possible effects of C-truncated pRB expressed from the residual variant transcripts. As REC-I variants can differ by the position of the PTC, mutant pRBs differ by the extent of regions still present. For this reason, we investigated differences in phenotypic expression among REC-I variants regarding the position of the truncation within the pRB structure. Patients with a truncation of pRB in the linker region between RB-N and pocket A showed a very low age at diagnosis and always developed bilateral disease. We analyzed if this finding for this specific linker region withstands if generalized to all unstructured linker regions. We found that the phenotypic expression in patients with PTCs in unstructured linker regions was apparently distinct from those with PTCs in structured domains (RB-N, pocket A, and pocket B). Because of its origin in explorative data analysis, the finding of a more severe phenotype in patients with PTCs in unstructured linker regions is to be regarded as tentative until validated by analysis of independent data sets. One approach is collaborative studies analogous to the strategy of meta-analyses that have aided in obtaining reproducible findings in genome-wide association studies [55]. As an explanation for our observation, we suggest that residual truncated pRB has an effect on tumorigenesis either via the normal pRB or on the normal pRB (i.e., in *trans*) prior to loss of the other allele and that this effect depends on the location of the truncation.

Previous studies have suggested that age of onset and incidence of SPM in individuals with heritable retinoblastoma are influenced by the type of pathogenic *RB1* allele, and risk is highest in children with variants that are regularly associated with complete penetrance for retinoblastoma [25,26]. Analysis of risk to SPM is complicated because external factors, especially treatment with external beam radiotherapy (EBRT), have a strong impact on the incidence. It was postulated that the impact of EBRT is most detrimental for children that receive EBRT in the first year of life [56]. The relative proportions of the variants in different REC classes in children with retinoblastoma vary by age at diagnosis. Specifically, compared to average, the percentage of REC-I variants is higher in children with an early diagnosis under the age of one year, while REC-III variants are less frequent. This age-dependent heterogeneity of variant effects may contribute to the effect of age at EBRT on the incidence of SPM and thus act as a confounder.

Collaborative studies as proposed above are also required to improve the classification system proposed here. Some but not all variants of type REC-III have been observed in individuals with incomplete penetrance. This heterogeneity may reflect different functional consequences of the different types of structural changes confined to regions of the pRB and suggests that deeper knowledge about the effect of REC-III variants may allow for subdividing REC-III into further subclasses. In our cohort, the number of patients with REC-III variants was too small to analyze this heterogeneity further. Analysis of the phenotypic expression of different REC-III variants in collaborative data sets is needed to characterize subclasses within REC-III.

Further improvements are also needed to better define the subgroup of variants in REC-I and REC-III with leaky effect. We had introduced these subclasses based on previous concepts and chose the location of a splice-site alteration as a simple criterium. It is expected that leakiness of splicing due to localization outside of invariant splice signals will result in some normally spliced transcripts and thus in a milder phenotype compared to non-leaky variants. This differential phenotypic expression was confirmed for REC-I-non-leaky and -leaky variants and is in line with previous findings [3,46]. However, a milder phenotype was not apparent for REC-III-leaky compared to REC-III-non-leaky variants. This could be due to the smaller effect size and the low number of observations in our study group but also to phenotypic heterogeneity within this class. In conclusion, current knowledge about leakiness REC-I or REC-III variants does not allow a stratification that facilitates risk prediction in genetic counseling. Again, a collaborative effort to broaden the available data set can help. In addition, in-vitro analyses of the determinants and effect of leakiness of splice variants are required.

Possible limitations and biases in our study were respected. We did not address parent-of-origin effects, which have been observed in retinoblastoma [4,5,30]. The human *RB1* gene is imprinted, and because of this, the expression of alleles is distinct by parental origin, at least in several cell types [57]. The parental origin of a variant allele is a plausible modifier of the phenotype. However, the effect of parental origin is analyzed best by comparing its effect for variants that are identical otherwise. The data analysis was performed in a retrospective study cohort, and a selection bias cannot be excluded. Only patients with constitutional *RB1* variants that developed retinoblastoma were included in the study, and for this reason, *RB1* variants associated with milder phenotypes or without the development of retinoblastoma in each individual might be underrepresented. The distribution of type of *RB1* variants among the study group is similar to other reported cohorts [49,51], and we consider our cohort despite this potential underrepresentation of variants with milder phenotypes as representative. Despite the exclusion of known mosaic patients from the study, high-level mosaicism in blood DNA may be undetected and could explain a milder phenotype regardless of the type of variant. Variants in REC-IV and -V were too rare in our cohort for any analyses. More data from other national cohorts are needed to characterize the effect of this pRB alteration on phenotypic expression. We are aware that every classification is only a proxy and can never reflect the full complexity of the real-world situation. According to Knudson’s assumptions, penetrance and tumor number are functionally linked by the Poisson distribution, and the Poisson parameter also determines the distribution of laterality. The distribution of age at diagnosis is determined by at least three components, the time of initiation, the time to reach a stage that causes signs or symptoms, and the time interval of parents or health caregivers to recognize these symptoms and act on them. These components are under the influence of other factors such as the location within the eye or social factors such as access to the medical resources required for diagnosis [58]. Of these components, the time of initiation and the time needed to reach a size that, in principle, permits clinical diagnosis may reflect the biological characteristic relevant for phenotypic variation, and its effect is blurred by the variation of non-biological factors. It is possible to control for these non-biological variations by examination of a study cohort of children with familial retinoblastoma that participated in neonatal screening, but the numbers in this study cohort are even smaller, as shown in our cohort with 14.8% of children that participated in a screening program.

## 5. Conclusions

Heritable retinoblastoma is a rare disease, and genotype-phenotype correlations are complex. We propose a retinoblastoma variant effect classification (REC) to facilitate risk prediction for further retinoblastoma in the clinical setting for attending physicians, patients, and families. REC provides a conceptual framework to facilitate the comparison of genotype-phenotype correlations among cohorts and to broaden our understanding of retinoblastoma tumorigenesis. Validation of REC with data from our study cohort highlighted a more severe phenotypic expression in children with REC-I than in REC-II and REC-III. This was even more pronounced in patients with premature termination at amino acids in one of the linker regions of pRB. As an explanation, we postulate that truncated pRB could have a dominant-negative effect on regular pRB, and localization of truncation modulates this dominant-negative effect. If confirmed in independent data sets, REC may contribute to a better understanding of retinoblastoma tumorigenesis, better estimation of the recurrence risk, and personalized ophthalmological surveillance in all at-risk individuals; potentially even for surveillance protocols for SPM.

## Figures and Tables

**Table 1:** Retinoblastoma effect classes (REC).

	Effect on pRB		Type of gDNA Alteration	Examples
	Structure	Quantity		
REC-I	C-truncated	Less	Intragenic deletions Small variants (SNV or indels) ORF exons 2–25 Splice-site	Nonsense or Frameshift variant
*REC-I (leaky)*	C-truncated and no change	Less	Small variants (SNV or indels) Remote splice-site	Splice-site variant
REC-II	NA	Complete loss	Whole gene deletions Partial gross deletions including 5′ end	Whole *RB1* gene deletions
REC-III	Confined change	No change	Intragenic deletions Small variants (SNV or indels) ORF exons 1–25 Splice-site	Missense or in-frame SNV
*REC-III (leaky)*	Confined change and no change	No change	Small variants (SNV or indels) Remote splice-site	Splice-site variant
REC-IV	C-truncated	No change	Partial gross deletions including 3′-end Small variants ORF exons 25–27 (no NMD)	SNV or indels 3′ end
REC-V	No change	Less	Small variants regulatory promoter motifs	SNV in promotor region

NMD, nonsense-mediated decay; ORF, open reading frame; SNV single nucleotide variant.

**Table 2:** Patient characteristics.

Clinical Characteristics	Study Cohort
**All**	287
**Sex**	
Female	137
Male	149
Unknown	1
**Laterality**	
Unilateral	32
Bilateral	255
**ICRB Group of 415 of 542 affected eyes**	
ICRB A	48
ICRB B	147
ICRB C	27
ICRB D	46
ICRB E	147
Missing information	127
**Family history**	
Isolated	245
Familial	39
Unknown	3

^1^ nomenclature according to http://varnomen.hgvs.org/recommendations/DNA/variant/delins/ (accessed on 28 March 2021). ICRB, International classification of retinoblastoma

**Table 3:** Classification of patients from the study cohort into the retinoblastoma variant effect classification.

REC Class	*n* (%)	Laterality *n* (% Within Laterality)		Age at Diagnosis (Months)		Number of Tumors in the Less-Affected Eye at Diagnosis of Each Patient	
		Unilateral	Bilateral	Median	Range	Median	Range
REC-I	199 (69.3)	13 (6.5)	186 (93.5)	7.3	0.2–48.0	2	0–8
*REC-I-non-leaky*	190 (66.2)	11 (5.8)	179 (94.2)	7.2	0.2–48.0	2	0–8
*REC-I-leaky*	9 (2.8)	2 (22.2)	7 (77.8)	12.0	2.5–19.3	1	0–2
REC-II	39 (13.6)	9 (23.1)	30 (76.9)	10.3	0.4–40.9	1	0–3
*REC-II*−	6 (2.1)	/	6 (100)	11.0	2.6–27.8	1	1–2
*REC-II+*	27 (9.4)	8 (29.6)	19 (70.4)	8.4	0.4–40.9	1	0–2
REC-III	45 (15.7)	9 (20.0)	36 (80.0)	11.6	0.9–45.1	1	0–4
*REC-III-non-leaky*	39 (13.6)	9 (23.1)	30 (76.9)	12.3	0.9–45.1	1	0–4
*REC-III-leaky*	6 (2.1)	/	6 (100)	8.2	4.1–14.6	1	1–4
REC-IV	1 (0.3)	0	1 (100)	13.1	/	2	/
REC-V	3 (1.0)	1 (33.3)	2 (66.7)	18.4	18.0–34.8	1	0–2
**Total**	287 (100.0)	255 (88.9)	32 (11.1)	8.3	0.2–48.0	1	0–8

REC, rb variant effect classification.

**Table 4:** Phenotypic expression of *RB1* variants with observed association to incomplete penetrance (incomplete penetrance) and without such observations (regular penetrance).

Feature of Phenotypic Expression	Regular Penetrance (*n* = 240)	Incomplete Penetrance (*n* = 47)
Age of diagnosis		
Median	8.0 months	12.4 months
Range	0.2–48.0 months	0.4–40.9 months
Number of tumors in the less-affected eye at diagnosis per patient		
Mean	2	1
Range	0–8	0–4
Laterality		
Unilateral (in % per column)	15 (6.3%)	17 (36.2%)
Bilateral (in % per column)	225 (93.8%)	30 (63.8%)

## Data Availability

Aggregated data are available on personal request to the authors.

## Appendix A

**Table A1:** List of all variants with observations of incomplete penetrance.

Variant Description ^1^	Type of *RB1* Variant	Retinoblastoma Effect Class	Observations of IP
Contiguous gene deletions including MED4	Whole *RB1* deletion	REC-II	Multiple i.e., Mitter et.al. 2011 [19] (model proposed by Dehainault et al. [20])
c.19dup	Frameshift PTC exon 1	REC-III	1 of 3 families with IP (2 unaffected)
c.54_79del	Frameshift PTC exon 1	REC-III	2 unaffected in one family
c.67_104del	Frameshift PTC exon 1	REC-III	2 unaffected in one family
c.1439_1441del	In-frame	REC-III	2 families with IP published and unpublished Lohmann et al. 1994 [27]
c.1981C > T	Missense	REC-III	18 families with IP
c.2116_2118dup	In-frame	REC-III	3 relatives with IP in one family, unpublished
c.2211 + 1G > T	Splice-site	REC-III	1 of 4 families with IP (1 unaffected)
c.2171T > G	Missense	REC-III	2 relatives with IP in one family, unpublished
c.2717_2718dup	Frameshift PTC Exon 27	REC-III	Mitter 2009 [59]
c.-198G > A	Promoter	REC-V	Taylor et al. 2007 [53] Two families, more than 2 ip
c.658C > G	Splice-site	REC I-leaky	Two families with 1 IP, one published in Zhang et al. 2008 [46]
c.1695 + 6T > C	Splice-site	REC I-leaky	One family with 6 IP
c.939G > A	Splice-site	REC III-leaky	One family with 1 IP

^1^ Nomenclature small variants according to Human genome variation society based on reference transcript LRG_517t1. IP incomplete penetrance.
